# Systemic loxoscelism with hemolysis and positive Coombs: diagnostic and physiopathological implications

**DOI:** 10.1590/S1678-9946202668058

**Published:** 2026-08-21

**Authors:** Júlia Alcântara Costa, Leticya Ribeiro Rocha, Adebal de Andrade, Cecilia Maria de Souza Lagares Dabien Haddad, Juliana Sartorelo Almeida

**Affiliations:** 1 Fundação Hospitalar do Estado de Minas Gerais, Hospital João XXIII, Centro de Informação e Assistência Toxicológica de Minas Gerais, Belo Horizonte, Minas Gerais, Brazil. E-mail: jsartorelo@gmail.com; 2 Fundação Hospitalar do Estado de Minas Gerais, Hospital João XXIII, Departamento de Pediatria, Belo Horizonte, Minas Gerais, Brazil

**Keywords:** Loxosceles, Brown spider, Hemolytic anemia

## Abstract

Brown spider (*Loxosceles*) envenomation is a critical public health issue in Brazil, the systemic cutaneous-hemolytic form of which represents a rare but life-threatening complication. This manifestation is primarily driven by sphingomyelinase D, a potent toxin that triggers a complex cascade of direct erythrocyte membrane disruption and complement-mediated destruction. While systemic cases are more frequently documented in younger populations, reports in octogenarians are exceptionally rare, posing significant diagnostic and therapeutic challenges. This study details a rare case of cutaneous-hemolytic loxoscelism in an 82-year-old woman. Initially misdiagnosed with a primary skin infection, the patient presented four days post-injury with a 6-cm necrotic lesion, fever, and anemia. Laboratory evaluation confirmed a significant hemoglobin drop, elevated lactate dehydrogenase, and a strongly positive direct antiglobulin test (4+), indicating immune-mediated hemolysis. Management included specific antivenom and high-dose corticosteroid therapy (prednisone), leading to clinical stabilization without the need for blood transfusion. The patient was monitored in the intensive care unit with conservative wound management. This report emphasizes that prompt recognition and multidisciplinary intervention are vital for favorable outcomes in rare, high-risk geriatric presentations with prominent synergy between toxic effects and immune dysregulation.

## INTRODUCTION

*Loxosceles*, popularly known as the brown spider, is the spider genus of greatest epidemiological importance in Brazil. From 2017 to 2021, it caused 39,409 accidents, accounting for 23.40% of all identified spider accidents in the country^1^. During the same period, 37 deaths were attributed to this genus, representing 40.22% of all spider-related deaths, with an estimated fatality rate of 0.09%^1^. Data from the Brazilian Notifiable Diseases Information System recorded 44,544 spider-related accidents in 2024. Among these, Loxosceles envenomation accounted for 9,900 cases (approximately 22.2%), resulting in 21 confirmed deaths associated with this genus^2^.

The brown spider is a small arachnid, approximately 3.5 cm in leg span, with long, thin legs and six eyes in three pairs, forming a crescent moon pattern^2^. In Brazil, accidents predominantly occur in its Southern Region, with a seasonality marked by higher incidence during the warmer months of the year^1^. The species occurs in indoor (intradomicile) and outdoor (peridomicile) environments^3^.

The bite of this non-aggressive spider is generally minimally painful, occurring most frequently when the animal is pressed against the body, such as individuals are dressing themselves or putting on their shoes^2^. Therefore, accident diagnosis commonly occurs retrospectively based on the evolution of the skin lesion as patients often fail to perceive the moment of the bite^3^.

Most accidents appear as a local skin lesion. The most typical lesion is called a marmoreal plaque, characterized by areas of blisters and ecchymoses interspersed with regions of ischemic pallor surrounded by an erythematous halo that progresses to dry necrosis. A diffuse skin rash may be present^3^. A less common and severe form of presentation is cutaneous-hemolytic loxoscelism, associated with the development of hemolysis and acute renal failure^3^ ^), (^ ^4^.

Extensive research across major databases (Cochrane, LILACS, SciELO, MEDLINE, PubMed, PMC) suggests that hemolytic anemia associated with a *Loxosceles* bite is a rare event in older adults, being predominantly reported in children and young adults^4^ (median age of 14 years). The scientific literature on the subject has no exact number of systemic loxoscelism cases in older patients. Demographic data from various studies report median ages ranging from 9 to 14 years, with a vast majority of cases occurring in patients under the age of 30 years^4^ ^), (^ ^5^ ^), (^ ^6^ ^), (^ ^7^. While individual case reports of severe or fatal outcomes focus on patients aged up to 54 years, the only noted exception is a Brazilian study involving patients up to 75 years of age that found no direct correlation between age and hemolysis. However, even that study lacks specific details regarding the number of older patients who developed systemic symptoms^6^.

This study aims to describe the case of an older patient who developed cutaneous-hemolytic loxoscelism associated with a positive direct Coombs test and to discuss the peculiarities and controversies related to its hematological diagnosis.

### Ethics

This case report was submitted to and approved by the ethics committee of Fundacao Hospitalar do Estado de Minas Gerais, Hospital Joao XXIII. It is registered on Plataforma Brasil under CAAE nº 95193226.5.0000.5119.

## CASE REPORT

**Figure 1:**
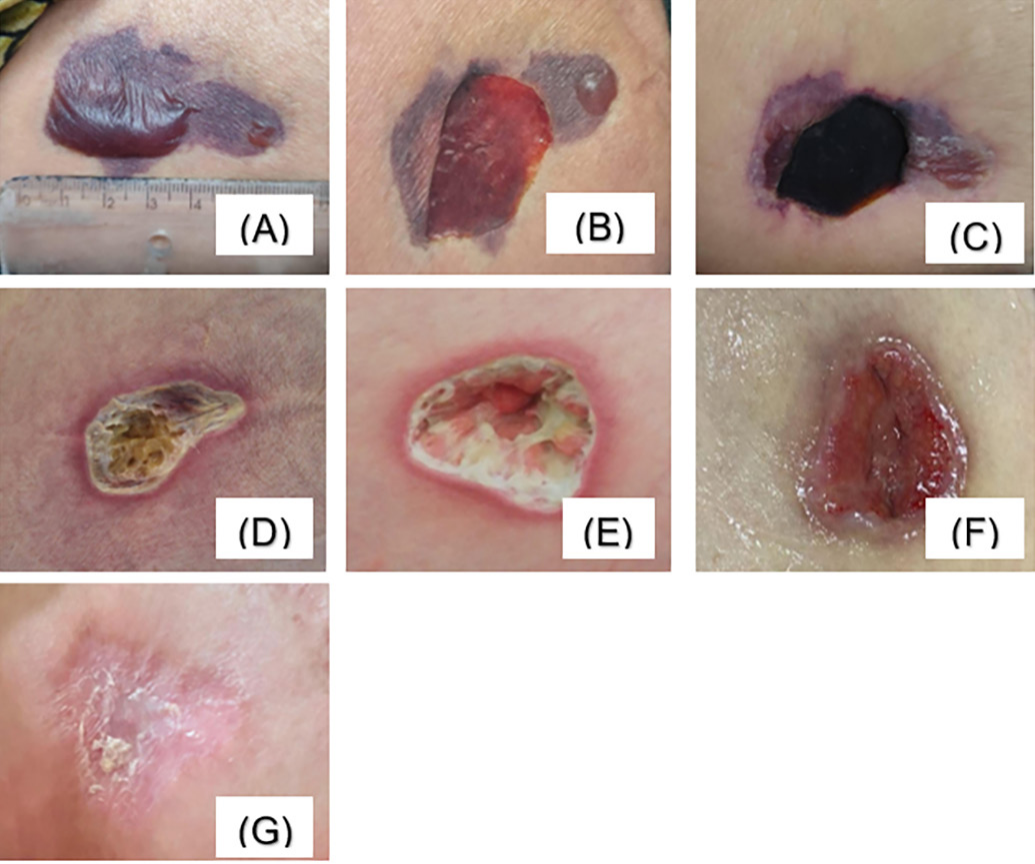
Chronology of the lesion: (A) Injury at admission with 3 days, (B) Lesion with 4 days, (C) Lesion with 5 days, (D) Lesion with 12 days, (E) Lesion with 35 days, (F) Lesion with 65 days, (G) Lesion with 112 days.

An 82-year-old female patient weighing 60 kg with hypothyroidism, systemic arterial hypertension, and dyslipidemia began to experience itching in the lateral region of her right thigh with no apparent lesion. The patient initially sought emergency care 24 h after the lesion appeared, complaining of intense local pain and purplish skin discoloration. Although laboratory tests revealed leukocytosis, the attending physician suspected a primary skin infection, prescribing symptomatic treatment and antibiotics. However, four days after this initial evaluation, the patient was admitted to a toxicology reference center due to clinical worsening. At that time, she presented with a dark purplish, infiltrated lesion measuring approximately 6 cm in diameter with irregular borders, a heterogeneous surface, and a blister containing rheumatic fluid, along with a skin rash on her torso and the medial aspect of her upper limbs (Figure 1). Further laboratory evaluation showed hemolysis (elevated LDH and indirect bilirubin) (Table 1). Given this clinical picture, a diagnostic hypothesis of cutaneous-hemolytic loxoscelism was raised: 10 vials of anti-arachnid serum were administered and prednisone (60 mg/day) was initiated.

**Table 1:** Comparative laboratory data during the patient’s stay at the reference center.

TEST Reference interval (unit)	D1*	D4	D5	D6**	D13	D14	D16***	D23
Hemoglobin 12.0-15.5 (g/dL)	11.5	7.1	-	5	7.5	7.5	7.5	9.8
Hematocrit 36-44 (%)	36	21.6	-	16.2	24.5	24.8	26.5	31.6
Total WBC Count 4.500-11.000 (x 10³/µL)	6730	30750	-	35490	11260	12020	7850	8210
Platelets 150.000-450.000 (x 10³/µL)	105000	173000	-	219000	174000	189000	198000	198000
Reticulocytes 0.5-1.5 (%)	1.1	2.7	-	-	-	14.3%	-	-
INR 0.8-1.1	1.17	1	-	1.06	-	-	-	-
Prothrombin Activity 70-100 (%)	80%	100%	-	-	-	-	-	-
aPTT 25-35 (seconds)	26	18.4	-	17.7	-	-	-	-
Urea 15-45 (mg/dL)	82	76	-	79	25	27	31	50
Creatinine 0.6-1.1(mg/dL)	1.31	0.88	-	0.75	0.74	0.73	0.75	0.77
Total Bilirubin 0.3-1.2 (mg/dL)	2.27	3.54	-	-	-	-	-	-
Indirect Bilirubin 0.2-0.8 (mg/dL)	1.43	3.19	-	-	-	-	-	-
Direct Bilirubin 0.0-0.3 (mg/dL)	0.84	0.35	-	-	-	-	-	-
Lactate Dehydrogenase 140-280 (U/L)	265	406	-	665	-	-	-	-
Serum Iron 50-170 (µg/dL)	-	-	163	-	52	-	-	-
Ferritin 10-120 (ng/mL)	-	-	2120	-	667	-	-	-
Haptoglobin 30-200 (mg/dL)	-	-	<20	-	-	-	-	-
Transf. Saturation (%) 15-50 (%)	-	-	82%	-	21%	-	-	-
Vitamin B12 200-900 (pg/mL)	-	-	696	-	-	-	-	-
Folic Acid >3.0 (ng/mL)	-	-	4.6	-	-	-	-	-
Bicarbonate 22-28 (mEq/L)	-	-	-	21.6	21.9	22.3	23.7	25.4

-=Test not performed on these dates; *D1 (Day 4 of symptom onset): Initiated prednisone 1 mg/Kg/day and administered 10 vials of antivenom; **D6: Antibiotic switch; ***D16: Prednisone dose increased to 2 mg/kg/day. WBC: White Blood Cell count; INR: International Normalized Ratio; aPTT: Activated Partial Thromboplastin Time.

During hospitalization, she developed fever and worsening leukocytosis, leading to the reintroduction of amoxicillin combined with clavulanate. The skin rash worsened after the reintroduction of the medication (raising the hypothesis of an amoxicillin hypersensitivity reaction). Therefore, this medication was switched to ciprofloxacin and Clindamycin and maintained for seven days.

The patient was jointly monitored by the plastic surgery team, with slow and gradual improvement of the lesion during follow-up. Blood cultures remained negative.

The patient experienced a progressive drop in hematimetric levels, associated with postural hypotension and fatigue, requiring monitoring in a semi-intensive care unit. A hematology consultation was requested, and additional tests for hemolysis investigation were performed on the fifth day of hospitalization, which showed a highly positive direct Coombs test (4+) and suppressed haptoglobin, consistent with immune-mediated hemolytic anemia secondary to loxoscelism. Ivermectin prophylaxis was administered, and the prednisone dose was increased to 120 mg/day for 14 days. The patient showed gradual improvement in hemoglobin levels.

The patient was discharged 12 days after admission, with instructions for gradual prednisone tapering and outpatient follow-up with hematology to combat anemia and with plastic surgery for the necrotic lesion.

## DISCUSSION

The reported case illustrates a severe systemic complication in an older patient resulting from a *Loxosceles* bite. The main venom components associated with local and systemic toxicity include hyaluronidases, sphingomyelinase D, collagenases, and phosphodiesterases^4^ ^), (^ ^5^. Although systemic manifestation is rare and more commonly observed in individuals below 20 years of age, it reflects an exacerbated immune response, which can be considered a form of immune-mediated hemolysis, as in the described patient^5^ ^), (^ ^6^ ^), (^ ^7^.

**Figure 2:**
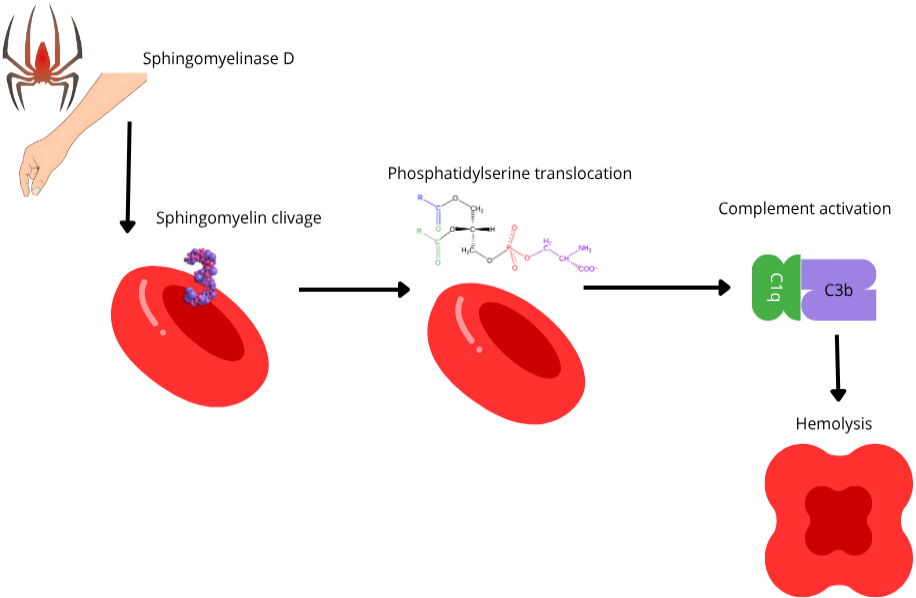
Pathophysiology of hemolysis with positive direct Coombs in systemic loxoscelism. Mechanism of hemolysis induced by *Loxosceles* venom sphingomyelinase D. The illustration details the pathophysiological cascade initiated by a spider bite. The enzyme sphingomyelinase D cleaves sphingomyelin in the erythrocyte membrane, translocating phosphatidylserine. This process activates the complement system (C1q and C3b), culminating in the hemolysis (destruction) of red blood cells.

The best-understood pathophysiology of hemolytic anemia in loxoscelism is predominantly mediated by the action of sphingomyelinase D^8^ ^), (^ ^9^ ^), (^ ^10^ ^), (^ ^11^. According to Robinson *et al.* ^4^, the pathogenesis of hemolysis in systemic loxoscelism is multifactorial. Beyond immune-mediated destruction, anemia may result from direct toxin-mediated erythrocyte damage, in which sphingomyelinase D interacts with membrane lipids to cause immediate structural destabilization independent of antibodies. This enzyme cleaves sphingomyelin, a phospholipid in the erythrocyte membrane, structurally changing the cell surface. Consequently, it externalizes phosphatidylserine, normally restricted to the inner membrane layer, to the outer face of the erythrocyte, changing the shape of the red blood cells^7^. The exposure of phosphatidylserine favors the activation of the classical complement system pathway via direct C1q binding to the erythrocytic membrane, which can lead to intravascular hemolysis and promote morphological alterations, such as spherocyte and stomatocyte formation (Figure 2)^7^ ^), (^ ^8^ ^), (^ ^9^ ^), (^ ^10^ ^), (^ ^11^.

In parallel, the deposition of complement fragments, especially C3b, on the erythrocyte membrane facilitates their opsonization and subsequent removal by splenic and hepatic macrophages, characterizing extravascular haemolysis^9^ ^), (^ ^12^.

The high prevalence of positive direct antiglobulin tests in severe cases confirms the immune component, yet the intensity of the hemolytic process suggests that direct cytotoxic effects also serve as a critical contributing factor.

Additionally, systemic loxoscelism can trigger an immune response that can induce the production of significant quantities of IgG class autoantibodies^7^. Red blood cells exposed to brown spider venom show deposition of IgG, in addition to complement system components, on their surface^8^ ^), (^ ^11^ ^), (^ ^12^.

The Coombs test (a direct antiglobulin test) identifies antibodies or complement bound to red blood cells via antiglobulin-induced agglutination^13^. In this case, the gel column method (anti-IgG/complement) was used, which confirms their presence but is unable to differentiate between IgG and complement^13^.

The positivity of the direct Coombs test in hemolytic anemia induced by *Loxosceles* venom reflects the adherence of complement components (such as C1q or C3b) or immunoglobulins to the erythrocyte surface. The global incidence of positive direct Coombs tests in loxoscelism remains unknown. However, positivity occurs in more than half of the patients with severe forms of the disease^6^ ^), (^ ^7^ ^), (^ ^14^. Furthermore, some negative results may correspond to false negatives as the quantity of antibodies bound to red blood cells might be below the detection limit of available commercial reagents^7^ ^), (^ ^9^ ^), (^ ^10^.

Despite the patients’ evolution time, antivenom serotherapy was recommended due to the presence of haemolysis^2^ ^), (^ ^3^ ^), (^ ^6^ ^), (^ ^14^. The appropriate antimicrobial regimen, coupled with the introduction and subsequent adjustment of prednisone dosage, proved to be crucial in controlling the infectious process and the systemic inflammatory response^14^. Approximately 2%-3% of *Loxosceles* accidents also present associated bacterial infection, and, in these cases, the diagnosis is confirmed by the association of worsening local appearance and constitutional symptoms, such as fever and asthenia^14^. However, note that leukocytosis in systemic loxoscelism often stems from the intense systemic inflammatory response triggered by the sphingomyelinase D in the venom^6^ ^), (^ ^14^ ^), (^ ^15^. This enzyme induces the release of pro-inflammatory cytokines, which can mimic a septic clinical picture-including fever and significant white blood cell elevation-even in the absence of a confirmed bacterial process^4^ ^), (^ ^6^ ^), (^ ^14^.

In Brazil, anti-*Loxosceles* or anti-arachnid sera (which contains antivenom fractions for *Loxosceles*, *Phoneutria*, and *Tityus*) constitute the first-line treatment for both, cutaneous lesions presenting within 36 h, and its cutaneous-hemolytic form^11^ ^), (^ ^12^ ^), (^ ^13^ ^), (^ ^14^ ^), (^ ^15^ ^), (^ ^16^ ^), (^ ^17^. The treatment regimen recommended by the Ministry of Health classifies envenomation into cutaneous (mild, moderate, severe) and cutaneous-visceral forms based on lesion size and systemic involvement. Symptoms range from small, painless lesions in mild cases to extensive necrosis, generalized edema, and hemolysis in severe or visceral forms. Treatment scales from symptomatic outpatient monitoring to oral corticosteroids and intravenous anti-venom serum depending on clinical severity^3^.

Corticosteroids constitute an important adjuvant treatment for immune-mediated hemolysis, with prednisone administration recommended at a dose of 1 to 2 mg/kg/day, maintained until hemoglobin exceeds 10 g/dL, with subsequent gradual reduction to prevent relapses^2^ ^), (^ ^3^ ^), (^ ^5^ ^), (^ ^6^ ^), (^ ^14^. Although prednisone use in loxoscelism is common practice, further studies are needed to more precisely define the ideal dose and duration of the treatment.

Recent research aimed at mitigating hemolysis associated with loxoscelism highlights eculizumab-a complement system inhibitor-as a promising therapeutic strategy, with favorable *in vitro* results^5^ ^), (^ ^9^.

In this reported case, multidisciplinary follow-up with the participation of the Hematology and Plastic Surgery teams was essential to ensure the patient’s full recovery.

Although rarely reported, the literature has described systemic loxoscelism with secondary immune-mediated hemolysis, which occurs mainly in young patients^4^ ^), (^ ^6^ ^), (^ ^10^ ^), (^ ^14^. However this case included an older patient. Advanced age constitutes a significant risk factor for a worse clinical prognosis in loxoscelism^12^. In older patients, the predisposition to severe outcomes can be linked to a higher prevalence of chronic comorbidities-such as cardiovascular diseases, diabetes, and obesity-which can exacerbate the systemic inflammatory response^14^. Advanced age is a primary demographic for autoimmune hemolytic anemia in general, and the presence of underlying autoimmune comorbidities-such as hypothyroidism or other immunologic disorders-serves as a critical risk factor for the development of hemolytic anemia^18^. Thus, when an older patient with a complex autoimmune profile suffers a *Loxosceles* bite, the intersection of senile physiological changes and prior immune dysregulation may create a heightened vulnerability to severe systemic complications.

## CONCLUSION

This case emphasizes the need for rigorous clinical vigilance and improved treatment guidelines for loxoscelism given its potential for progression to severe cases. This highlights the importance of understanding associated systemic manifestations, such as autoimmune hemolytic anemia, when dealing with *Loxosceles* envenomation.

Assistance protocols suggest greater efficacy of specific serotherapy at any time for the cutaneous-hemolytic form at a full dose^2^ ^), (^ ^3^ ^), (^ ^12^.

## Data Availability

The anonymized dataset in this study is available from the corresponding author upon reasonable request.
